# The Awareness of Methylphenidate and Its Use: Experiences and Perceptions of Medical Students

**DOI:** 10.7759/cureus.74317

**Published:** 2024-11-23

**Authors:** Burak Onal, Melik Yigit Bayindir, Yasemin Begum Topkarci, Aslihan Seyda Dogan, Burhaneddin Oktan, Oruc Yunusoglu

**Affiliations:** 1 Medical Pharmacology, Biruni University Faculty of Medicine, Istanbul, TUR; 2 Medical Pharmacology, Istanbul University Cerrahpasa Faculty of Medicine, Istanbul, TUR; 3 Medical Pharmacology, Bolu Abant İzzet Baysal University Faculty of Medicine, Bolu, TUR

**Keywords:** addiction, attention deficit hyperactivity disorder, medical school students, methylphenidate, misuse

## Abstract

Background and objective

Over the past decade, the use of psychostimulants typically prescribed for attention-deficit/hyperactivity disorder (ADHD), such as methylphenidate (MP), has become popular among undergraduate students to enhance their academic performance. Despite potential health and legal repercussions, the misuse of these medications has become a significant public health issue, not only in the general population but particularly among students in medical schools across Turkey. This study investigated the prevalence of MP misuse among Turkish medical students and the factors contributing to it.

Methods

We conducted a survey involving 418 medical students (257 female, 161 male), covering addiction history, physician-recommended ADHD medication, sharing and recommending MP among peers, initial exposure to MP misuse information, reasons for non-prescription MP use, duration of misuse, perceptions of MP's addictive potential, and ethical views on MP use to help with exams.

Results

The findings revealed that senior students showed a higher level of awareness and earlier initiation of MP misuse compared to younger students (p<0.05). Residing in student housing emerged as a significant reason for exposure to and subsequent misuse of non-medical prescription MP (p<0.05). Both MP misusers and non-users acknowledged the MP's addictive nature. Peer influence was the primary factor for initial recommendations of MP misuse (p<0.05).

Conclusions

While academic achievement appears to be the primary motivator for MP misuse, the effectiveness of this practice in non-ADHD students is uncertain. Implementing proactive measures is crucial to curb such misuse, particularly among medical students, to prevent a future global health concern.

## Introduction

Substance use disorders, such as those involving psychostimulants, are complex and multifaceted problems that negatively change the quality of life not only for misusers but also for their family and society at large. Psychostimulant use disorders are a significant public health problem worldwide [1,2]. Substantial efforts have been made to develop pharmacological, psychosocial, and community-based treatments [3]. Despite these efforts, according to global estimates, only one in six or fewer individuals in need of substance use disorder treatment receive it [3,4]. While illicit substances have long been a societal concern, substance use rates have been on the rise in recent years. According to global surveys, an estimated 4.9 million people worldwide have amphetamine use disorder [5].

Attention-deficit/hyperactivity disorder (ADHD) was first described in 1902. ADHD, which typically occurs during childhood, is characterized by excessive hyperactivity and/or inattention and executive dysfunction, as well as impulsivity and a lack of motivation and emotional self-control [2]. Individuals with a family history of ADHD reportedly have a 10 times or higher risk of developing the disorder. The prevalence of ADHD is reported to be 2.5-4.9% in adulthood, whereas it is approximately 5.3% among children [6]. ADHD is considered a chronic disorder, and approximately 30-50% of those diagnosed during childhood carry its core symptoms into adulthood [7]. Both pharmacotherapy and behavioral therapy are used in the treatment of ADHD. However, behavioral therapies are not widely available due to a lack of experts. Hence, only pharmacotherapy is used in most ADHD treatments [6].

Psychostimulants have been used to treat ADHD since 1937 and are approved by the Food and Drug Administration (FDA) for use in individuals aged six years and older [8]. The 1971 Convention on Psychotropic Substances categorized ADHD stimulants as Schedule II prohibited substances in the US due to their potential for misuse, dependency, and harm to both individual and public health [9]. Pharmacotherapy options for ADHD in adults include both psychostimulant and non-stimulant options. The availability of individual pharmacotherapies differs by region and jurisdiction, and likewise by treatment guidelines. Guidelines in Turkey recommend long-acting methylphenidate (MP), amphetamine mixture, or lisdexamfetamine as first-line pharmacological options, with short-acting MP or atomoxetine or dextroamphetamine as second-line options for patients with inadequate response to first-line treatments. These guidelines suggest psychostimulants as first-line treatment for adults, with guanfacine or atomoxetine considered as first-line treatment in some clinical situations [10].

MP (sample brand names: Metadate, Ritalin, Focalin, Concerta, Methylin) is available as an orally disintegrating tablet, liquid, or in capsule form. Additionally, MP is approved by the FDA as a second-line treatment for narcolepsy in adults. Off-label uses of MP include treatment for apathy in Alzheimer's disease, enhancing cognitive performance, fatigue in patients with cancer, and refractory depression in the geriatric population [11]. MP elevates extracellular dopamine by inhibiting the dopamine transporter. It is also a noradrenaline re-uptake inhibitor with agonist-like effects [12]. These mechanisms underlie both its therapeutic and addictive effects [7,13]. MP shares pharmacological properties with well-known drugs with a high potential for addiction, such as amphetamine and cocaine [14].

Concerns have been raised over the misuse potential of MP as it has been demonstrated to have similar drug-seeking reinforcing effects to cocaine. However, it is worth noting that MP has a lower potential for misuse than cocaine. This has been linked to a comparatively slow rate of brain clearance, which reduces the possibility of MP misuse and repeated dosing [14]. It is misused by adolescents and adults, and the diversion and misuse of ADHD drugs are serious clinical problems [7,13]. Preclinical studies suggest that drugs with high addiction potential in animals are more likely to be misused clinically [13]. Various scientific preclinical studies have demonstrated that MP causes addiction in animals [7].

It has been demonstrated that systemic MP functions as a reinforcer in the conditioned place preference and self-administration procedure in monkeys, mice, and rats [7,13]. Concerns have also been raised that treatment with MP for ADHD may lead to a greater propensity to drug and substance misuse [7]. Evidence from experimental exams indicates that MP injections can increase subsequent psychostimulant- (e.g., amphetamine and cocaine) conditioned place preference and self-administration [7,13]. According to more recent French research based on the 2008-2010 OPPIDUM survey, the majority of MP misusers (85.4%) were men, 70% came from relatively poor socioeconomic backgrounds, 68% got their MP illegally, and 52% used it intravenously [9].

MP is also often misused by high school and college students to enhance cognitive function or as a recreational substance. This off-label use of MP appears to be most prevalent among young adults aged 18-25 years, with existing data indicating that up to one-quarter of high school students engage in the non-medical use of prescription stimulants [15]. Students attending high schools with high rates of psychostimulant treatment for ADHD are 36% more likely to engage in non-medical use of prescription stimulants compared to those attending schools with the lowest academic achievement; this illustrates how exposure to treatment and academic pressure may influence the misuse of medication [15,16]. Unfortunately, illegal MP misuse seems to have increased dramatically over the past several years [16].

Evidence indicates that medical students may have a higher propensity for medication misuse compared to students in other disciplines [17,18]. With the demanding changes brought about by different integrated curricula, these students often worry about underperforming academically and may feel immense pressure to succeed [17]. Other factors included weak parental influence, peer pressure, economic uncertainty, and stress [17,18,19]. This study aimed to determine the frequency of MP misuse among medical students in Turkey and the associated triggers that attracted them to such prescription drugs.

This article was previously presented as a meeting abstract at the 2nd International and 27th National Pharmacology Congress on November 24, 2023.

## Materials and methods

This was a cross-sectional study designed to determine the prevalence of therapeutic and non-therapeutic use of MP-containing medications among medical school students and their knowledge and attitudes toward related issues. Data were collected between May 17 and June 17, 2023, using an anonymous online survey prepared by the researchers based on their clinical experience and literature knowledge.

The study consisted of 871 students enrolled in the faculty of medicine at Biruni University in 2023. After the initial invitation to participate in the survey, weekly reminders were sent to all medical school students for four weeks. Due to its voluntary nature, the sample size was 418 students (47.99% of all Biruni University Medical School students) from the first to sixth grades of the faculty of medicine. The number of questions in the survey varied depending on the participants' answers. Depending on the direction of their answers, the participants answered between eight and 23 questions. Written informed consent was obtained from all participants. The survey did not include any questions that would reveal the identity of the participants. Participants were informed of their right to refuse to participate in the study or to withdraw from the study at any time.

The survey form included questions about demographic data, addiction status, previous ADHD diagnosis by a psychiatrist, medication recommendation for ADHD by a physician, status of medications used, MP user's recommendation and sharing of MP with friends, when and where they heard of MP, purpose of use if used without a doctor's recommendation, period of use, opinions about MP and addiction, and ethical evaluation of its use to improve exam performance. The complete survey is provided in the Appendices.

Ethical approval

Ethical approval for the study was obtained from the Biruni University Non-Interventional Clinical Research Ethics Committee (Decision No: 2023/81-04). The study was conducted by adhering to the Declaration of Helsinki.

Statistical analysis

Statistical analyses were performed using Jamovi software (version 2.4.11, The Jamovi Project, 2023). Descriptive statistics, including counts and percentages, were used to present qualitative data from the survey questions. To determine differences between the responses to the identified survey questions, appropriate tests such as Pearson's chi-square, Yates's corrected chi-square, likelihood ratio, and Fisher's exact test were applied. Differences among sub-responses of groups with significance were evaluated using the post-hoc Bonferroni test. The level of statistical significance was set at a p-value of 0.05.

## Results

**Figure 1:**
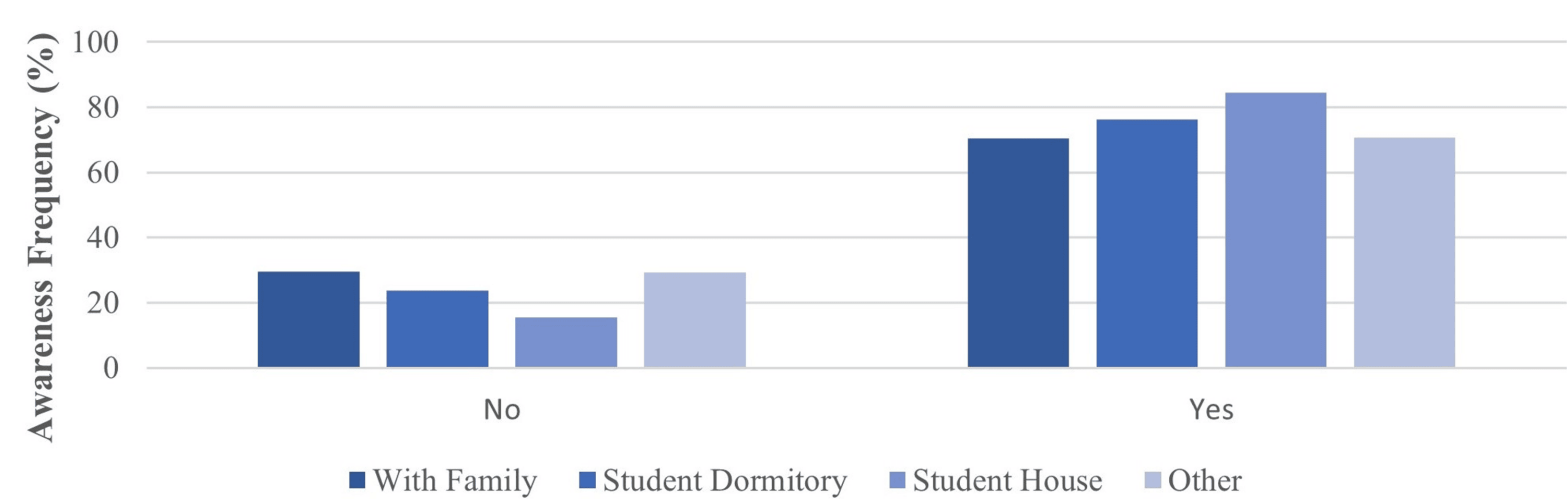
The association between methylphenidate awareness and place of residence status

Our analysis revealed significant associations between medical students' tendency to use MP and their academic or personal circumstances. A statistically significant association (p=0.05) was observed between the responses to the questions "Place of residence?" and "Have you heard of methylphenidate before?". Further analysis of place of residence revealed that students residing in student housing were statistically more likely to report awareness of MP compared to those living with family (p=0.035) (Table 1; Figure 1).

**Table 1:** The association between methylphenidate awareness and place of residence (n=418)

			Place of residence			
			With family	Student dormitory	Student house	Other
Have you ever heard of methylphenidate (Ritalin, Concerta, Medikinet) before?	No, n (%)	103 (24.64)	61 (29.46)	20 (23.80)	17 (15.45)	5 (29.41)
	Yes, n (%)	315 (75.35)	146 (70.53)	64 (76.19)	93 (84.54)	12 (70.58)
	Total, n (%)	418 (100.0)	207 (100.0)	84 (100.0)	110 (100.0)	17 (100.0)

Residence status (staying in a student house) also seems to attract the students to desire using MP since we found a positive correlation between the questions "Place of residence?" and "Does the fact that your friends are using methylphenidate and seeing its positive effects create a desire for you to use methylphenidate?" Residing in a student house appears to have a positive effect on students' tendency to use (p=0.011).

Also, the time they first heard the term “methylphenidate” may have a pivotal role in terms of using it. Those who heard the term for the first time at an older age had a negative correlation with the tendency to use it as we found a statistically significant difference between the responses to the questions "If you have not used methylphenidate, have you considered using it?" and "When did you first hear about methylphenidate?" There appears to be a trend of students encountering MP during high school. However, the proportion of these encounters is inversely proportional to students' age (p<0.001).

The year in medical school seems to determine the possible advisor who guides them through using MP. The year of medical school also changes whether their family, lecturer, or friends will tell them about methylphenidate. Chi-square analysis revealed a statistically significant association (p=0.008) between students' class year and their sources of information about methylphenidate, as shown by their responses to the questions "Which class are you in?" and "If you heard about it, from whom did you hear about methylphenidate?". Specifically, senior students were more likely to have heard about methylphenidate from clinical supervisors, while junior students primarily heard about it from peers or media sources.

The lower the class they were in, the higher the possibility they may hear about methylphenidate from their peers. A statistically significant difference was found between the responses to the questions "Which class are you in?" and "When did you first hear about methylphenidate?" (p<0.001) (Table 2).

**Table 2:** Distribution of student responses to the question "Do you think it is ethically acceptable to use methylphenidate to improve exam performance?" across medical school years (n=418)

			Which year are you in?					
			1	2	3	4	5	6
Do you think it is ethically acceptable to use methylphenidate to improve exam performance?	No, n (%)	235 (56.2)	51 (53.7)	35 (46.7)	47 (56.0)	30 (50.0)	34 (61.8)	38 (77.6)
	Yes, n (%)	183 (43.8)	44 (46.3)	40 (53.3)	37 (44.0)	30 (50.0)	21 (38.2)	11 (22.4)
	Total, n (%)	418 (100.0)	95 (100.0)	75 (100.0)	84 (100.0)	60 (100.0)	55 (100.0)	49 (100.0)

Year in medical school was inversely associated with the perceived ethical acceptability of using methylphenidate to enhance exam performance. The difference between the responses to the questions "Which class are you in?" and "Do you think it is ethically acceptable to use methylphenidate to improve exam performance?" were found to be significant (p=0.017) (Table 2).

We also found a positive correlation between gender, addiction tendency, and the time the students used methylphenidate. The difference between the answers to the questions “Your gender?’’ and “Are you addicted to anything?” (p<0.001), and ‘’Your gender?” and ‘’If you used methylphenidate, during which period did you use it?” (p=0.029) were found to be statistically significant (Table 3). Male students may exhibit a greater tendency to develop addictive behaviors related to MP use compared to female students. Interestingly, females demonstrate a pattern of regular MP use, while males exhibit a tendency towards increased MP use immediately preceding examinations (Table 3).

**Table 3:** Distribution of student responses to the question “If you have used methylphenidate, in which period did you use it?” across genders

If you have used methylphenidate, in which period did you use it?	Gender		
	Female	Male	Total
Regularly, n (%)	5 (83.3)	1 (16.7)	6 (100.0)
When the exam approaches, n (%)	0 (0.0)	4 (100.0)	4 (100.0)
During the exam, n (%)	4 (100.0)	0 (0.0)	4 (100.0)
While studying for the medical specialization exam, n (%)	0 (0.0)	1 (100.0)	1 (100.0
When the academic year begins, n (%)	2 (40.0)	3 (60.0)	5 (100.0)
Non-academic period, n (%)	3 (60.0)	2 (40.0)	5 (100.0)
Total, n (%)	14 (56.0)	11 (44.0)	25 (100.0)

The tendency to use methylphenidate without any medical recommendation may change according to the addiction status of the students and from whom they obtain methylphenidate. The difference between the answers to the questions ‘’Have you used methylphenidate without a doctor's recommendation?’’ and ‘’ Are you addicted to anything?” (p=0.005) and ‘’Have you used methylphenidate without a doctor's recommendation?’’ and ‘If you used methylphenidate without a doctor's advice, where did you obtain the drug?’’ (p=0.003) questions were found to be significantly different (Table 4). Our findings suggest that non-medical use of methylphenidate is associated with an increased risk of developing dependence. Students who engage in this practice may also be more likely to acquire the medication from peers rather than through authorized channels.

**Table 4:** Distribution of student responses to the question “If you used methylphenidate without a doctor's advice, where did you obtain the drug?” versus the question “Have you ever used methylphenidate without a doctor's recommendation?” (n=315)

		Have you ever used methylphenidate without a doctor's recommendation?		
		No	Yes	Total
If you used methylphenidate without a doctor's advice, where did you obtain the drug?	Friends, n (%)	97 (34.3)	21 (65.5)	118 (37.5)
	Doctor, n (%)	25 (8.8)	5 (15.6)	30 (9.5)
	Self-conducted research, n (%)	19 (6.7)	1 (3.1)	20 (6.3)
	Lectures, n (%)	105 (37.1)	3 (9.4)	108.4 (34.3)
	Social media, n (%)	17 (6.0)	0 (0.0)	17 (5.4)
	Documentaries, n (%)	2 (0.7)	0 (0.0)	2 (0.6)
	Other, n (%)	18 (6.4)	2 (6.3)	20 (6.3)
	Total, n (%)	283 (100.0)	32 (100.0)	315 (100.0)

Similarly, we found a statistically significant negative correlation between students' tendency to use methylphenidate without a medical recommendation and their belief about its addictiveness (p<0.001). Specifically, students who answered ‘’Yes’’ to the question ‘’Do you think methylphenidate is addictive?’’ were less likely to respond affirmatively to ‘’Have you ever used methylphenidate without a doctor's recommendation?’’. Conversely, students who adhere to prescribed use patterns may hold more accurate beliefs regarding the addictive properties of methylphenidate (Table 5).

**Table 5:** Distribution of student responses to the question “Have you ever used methylphenidate without a doctor's recommendation?” versus the question “Do you think methylphenidate is addictive?” (n=315)

		Have you ever used methylphenidate without a doctor's recommendation?		
		No	Yes	Total
Do you think methylphenidate is addictive?	No, n (%)	82 (29.0)	19 (59.4)	101 (32.1)
	Yes, n (%)	201 (71.0)	13 (40.6)	214 (67.9)
	Total, n (%)	283 (100.0)	32 (100.0)	315 (100.0)

The students who prefer to use methylphenidate to improve their exam performance seemed to use methylphenidate without a medical recommendation. There was a statistically significant difference between the responses to the questions ‘’Have you ever used methylphenidate without a doctor's recommendation?’’ and “Do you think it is ethically acceptable to use methylphenidate to improve exam performance?” (p=0.006). Students engaging in non-medical use of methylphenidate may exhibit a decreased perception of the ethical concerns surrounding its use for cognitive enhancement during examinations. Conversely, students who abstain from such practices may hold more stringent views on the acceptability of using methylphenidate to improve academic performance (Table 6).

**Table 6:** Distribution of student responses to the question “Do you think it is ethically acceptable to use methylphenidate to improve exam performance?” versus the question “Have you ever used methylphenidate without a doctor's recommendation?” (n=315)

		Have you ever used methylphenidate without a doctor's recommendation?		
		No, n (%)	Yes, n (%)	Total, n (%)
Do you think it is ethically acceptable to use methylphenidate to improve exam performance?	No, n (%)	165 (58.3)	10 (31.3)	175 (55.6)
	Yes, n (%)	118 (41.7)	22 (68.8)	140 (44.4)
	Total, n (%)	283 (100.0)	32 (100.0)	315 (100.0)

The tendency to use methylphenidate without a medical recommendation has a positive correlation with being in social circles where friends frequently discuss the perceived benefits of methylphenidate use. We found a statistically significant difference between the responses to the questions ‘’Have you used methylphenidate without a doctor's recommendation?’’ and “Does the fact that your friends are using methylphenidate and seeing its positive effects create a desire for you to use methylphenidate?" (p=0.007). Students engaging in non-medical use of methylphenidate may be more susceptible to peer influence, particularly from peers who themselves use methylphenidate and endorse its purported benefits.

## Discussion

The present survey seeks to explore the patterns and underlying factors associated with MP use in a population of medical school students. Additionally, it aims to evaluate existing concerns regarding the off-label application of MP as a cognitive enhancement. Stimulants have emerged as the primary pharmacological treatment for ADHD in recent decades [20]. These medications act by stimulating the central nervous system, leading to improvements in focus and concentration [21]. Interestingly, medications typically used for ADHD, a childhood-onset neurodevelopmental disorder characterized by hyperactivity, loss of attention, and impulsivity that can persist into adulthood and negatively impact professional, academic, and social life [22], are increasingly employed to enhance cognitive performance in healthy individuals. The timing of a college student's first encounter with MP may influence their awareness of this substance. According to our findings, residence status (staying in a student house) seems not only to attract the awareness level of MP but also to determine the student’s tendency to use MP if his/her MP-using friends keep talking about MP’s positive effects. The social circle may significantly influence a medical student's decision to use MP and their motivation to seek further information regarding its use.

Studies indicate that between 1.5% and 31% of college students diagnosed with ADHD receive treatment with stimulants such as MP [23]. MP users can be categorized into four distinct groups. Group 1 comprises individuals who lack an ADHD diagnosis and do not misuse prescription stimulants. Group 2 includes individuals with diagnosed ADHD who adhere to their prescribed medication regimen. Group 3 encompasses individuals without an ADHD diagnosis who misuse prescription stimulants. Finally, Group 4 consists of individuals with diagnosed ADHD who take their medication beyond the prescribed dosage. A growing body of research published within the last 10 years has shed light on the increasing concern regarding the non-medical use of psychostimulants among undergraduate students to improve academic performance [24]. This misuse can manifest in several ways, including taking stimulants without a prescription, using stimulants for reasons other than those prescribed, using stimulants to experience euphoria, or illegally selling or sharing a valid prescription [24].

Emerging evidence from initial investigations suggests a potential association between ADHD and a heightened risk of stimulant medication misuse among student populations [25]. However, in the present study, we found that senior medical students hold concerns regarding the ethical implications of using MP to enhance academic performance. Furthermore, many medical students, even those without prior experience with unsanctioned MP use, disapprove of its off-label application for cognitive improvement during examinations due to ethical considerations.

Students who engage in non-prescribed MP use primarily acquire the medication from friends within their social circles. Our findings revealed a positive association between students' year of study and their tendency to use MP following initial exposure. Social interactions, particularly those with friends and to a lesser extent family members and lecturers, may represent potential influences on medical students' tendency towards MP use. According to the present study results, senior medical students who engage in non-prescribed MP use primarily acquire the medication from friends within their social circles. Supporting these findings, in a well-designed survey, Wilens and Kaminski concluded that adolescents and young adults most frequently obtain stimulants from their peers [26]. This concerning pattern is further evidenced by the high prevalence of medication diversion (selling, trading, or giving away) reported among college students [27].

MP improves control of behavioral impulses and enhances focus. However, improper use of stimulants can lead to drug dependence [22]. According to our findings, a positive correlation may exist between non-prescribed MP use and the development of addiction among medical students. Moreover, we also found that both the frequency of MP use and drug addiction seem to be higher among senior medical students. Students who have used MP with and without a valid prescription both recognized the potential for addiction to the medication. The extent of MP misuse has been demonstrated to be a potential factor influencing students' susceptibility to addiction.

The term psychostimulant must be taken into account while using MP as it may have several side effects such as heart attack, increased heart rate, visual disturbances, anxiety, and decreased sleep quality [17,24]. The reasons behind medical school students' use of MP range from curiosity and weight loss to increased academic success; however, there are no supporting findings in the literature that confirm such performance enhancement in those without ADHD. In contrast, a substantial body of research suggests that psychostimulants, including MP, may not influence cognitive performance or academic achievement [28]. However, ia meta-analysis by Prasad et al. involving 43 studies reported an increase in productivity among users, with no corresponding improvement in accuracy [29].

Several methodological limitations warrant consideration when interpreting our findings. Firstly, the study employed a convenience sample with a small size, primarily consisting of medical school students from a single university. This limits the generalizability of our results to the broader population. Therefore, these findings should be considered preliminary until they can be replicated using more representative samples of medical school students in future studies. Secondly, the data collection relied on self-reported surveys, and no measures were taken to verify the accuracy of the information provided. While anonymity was assured, the sensitive nature of questions concerning prescription stimulant misuse might have led students to be hesitant in their responses.

## Conclusions

The misuse of prescription stimulants continues to emerge as a significant public health issue among the general population and, alarmingly, in medical schools. Strict guidelines should be put in place to guide the prescription of stimulants. A comprehensive medical and psychological evaluation is crucial before initiating treatment. This evaluation should consider factors such as childhood development, concurrent use of other psychoactive substances, academic performance, family history, and cognitive functioning. Further research is crucial to explore additional variables that contribute to the misuse of prescription stimulants among the medical school student population.

## Appendices

Evaluation of Methylphenidate Use of Medical Faculty Students: A Survey Study

You are cordially invited to participate in a research study titled "Evaluation of Methylphenidate Use Among Medical Students: A Survey Study." This study aims to investigate the patterns and motivations behind methylphenidate use among medical students, including its potential impact on academic performance and concerns about its use as a performance-enhancing substance.

Participation in this study is entirely voluntary. You have the right to decline participation without any repercussions. Your participation will not affect your current or future standing at the university. Your privacy is of utmost importance to us. The study will not collect any personal information that could identify you. All data collected will be kept confidential and stored securely. Your responses will be anonymized and used solely for research purposes.

The primary objectives of this study are to assess the prevalence and patterns of methylphenidate use among medical students, to investigate the motivations and perceived benefits of methylphenidate use among medical students, and to explore the potential impact of methylphenidate use on academic performance and concerns about its use as a performance-enhancing substance.

The study will employ a self-administered online survey to gather data from medical students. The survey will consist of questions about demographics, methylphenidate use history, motivations for use, perceived benefits and risks, and academic performance. The collected data will be analyzed using appropriate statistical methods to identify patterns and trends in methylphenidate use among medical students. The findings will be presented in a confidential report and may be published in peer-reviewed journals.

By completing the survey, you consent to participate in this research study. Your consent implies that you have read and understood the information and agree to participate voluntarily.

This study has been approved by the Biruni University Non-Interventional Clinical Research Ethics Committee under Decision No. 2023/81-04 and is conducted following the Helsinki Declaration.

If you have any questions or concerns about the study, please feel free to contact the researchers at Biruni University Faculty of Medicine.

1. Which class are you in?

1

2

3

4

5

6
 

2. What is your age?

3. What is your gender?

Female

Male

Other
 

4. Who do you live with? Where do you live?

With my family

With my friends in a student residence

With my friends in a student dorm

Other 
 

5. Are you addicted to anything? 

Yes, 

No (if your answer is No, Skip to Question 7)]
 

6. What are you addicted to?
 

7. Have you heard of methylphenidate (Ritalin, Concerta, Medikinet) before?

Yes, 

No (if your answer is No, Skip to Question 20)
 

8. From whom did you hear about methylphenidate?

From my friend, 

From my doctor, 

I researched it myself, 

From lectures, 

From social media, 

From documentaries, 

Other
 

9. When did you hear about methylphenidate?

Before high school, 

in high school, 

in the 1st year of medical faculty 

in the 2nd year of medical faculty 

in the 3rd year of medical faculty 

in the 4th year of medical faculty 

in the 5th year of medical faculty 

in the 6th year of medical faculty
 

10. Have you used methylphenidate without a doctor's recommendation?

Yes

No (if your answer is No, Skip to Question 19)
 

11. If you used methylphenidate without a doctor's recommendation, what were your intended use/expectations? (You can select multiple options.)

To keep me from falling asleep

To increase my academic success

For doping purposes in sports competitions

Because I'm curious about the effect

To lose weight

Other
 

12. If you used methylphenidate without a doctor's advice, where did you obtain the drug?

From friends 

From family members 

Other
 

13. If you used methylphenidate without a doctor's advice, when did you use it?

When the exam period approaches,

During the exam period,

While studying for the Medical Specialization Exam,

When the academic year begins,

During a period other than academic purposes
 

14. How did you feel after taking methylphenidate? (You can select multiple options.)

My learning performance increased,

My sleep has decreased,

My appetite has decreased,

I felt euphoric,

I was worried

I felt nervous,

I saw or heard things that didn't exist,

Other
 

15. If you have used methylphenidate, have you ever desired to use it again?

Yes during exam periods 

Yes when I feel sleepy 

Yes when I gain weight 

Yes but I did not use it 

Yes other 

No, because it is addictive 

No, because of other side effects 

No, because I could not obtain it 

Other
 

16. Have your drug expectations met as a result of using methylphenidate?

Yes

No

Partially Yes
 

17. If you used methylphenidate without a doctor's advice, did you recommend it to your friend?

Yes

No
 

18. Did you feel uncomfortable admitting to using methylphenidate?

Yes

No (whatever the answer is skip to question 20)
 

19. If you have not used methylphenidate, have you considered using it?

Yes 

No 

I used it for treatment purposes upon my doctor's recommendation.
 

20. Have you ever been diagnosed with Attention Deficit Hyperactivity Disorder (ADHD) by a psychiatrist?

Yes

No (if your answer is No, Skip to Question 32)]
 

21. How old were you when you were diagnosed with ADHD?
 

22. Has the doctor recommended any medication for ADHD? 

Yes

No (if your answer is No, Skip to Question 30)]
 

23. Have you used your recommended medicine?

Yes

No (if your answer is No, Skip to Question 29)]
 

24. Which medicine did you use?
 

25. What was your purpose/expectation for using methylphenidate? (You can select multiple options.)

To keep me from falling asleep

To increase my academic success

For doping purposes in sports competitions

Because I'm curious about the effect

To lose weight,

Other
 

26. If you used methylphenidate, during which period did you use it?

Regularly 

When the exam period approaches 

During the exam period 

While studying for the Medical Specialization Exam 

When the academic year begins 

During a period other than academic purposes

27. How do you feel after taking methylphenidate? (You can select multiple options)

My learning performance increased

My sleep has decreased

My appetite has decreased

I felt euphoric

I was worried

I felt nervous

I saw or heard things that didn't exist

Other
 

28. Are you still using Methylphenidate?

Yes,

No (Skip to question 30).
 

29. If you did not use methylphenidate, what is the reason?
 

30. If you were prescribed methylphenidate, did you recommend it to your friend?

Yes

No
 

31. If you were prescribed methylphenidate, have you ever shared your medication with your friend?

Yes

No
 

32. Do you think methylphenidate is addictive?

Yes

No
 

33. Does the fact that your friends are using methylphenidate and seeing its positive effects create a desire for you to use methylphenidate?

Yes

No
 

34. Do you think it is ethically acceptable to use methylphenidate for the purpose of improving exam performance?

Yes

No
